# One-Pot and Efficient Synthesis of Triazolo[1,2-a]indazole-triones via Reaction of Arylaldehydes with Urazole and Dimedone Catalyzed by Silica Nanoparticles Prepared from Rice Husk

**DOI:** 10.3390/molecules16119041

**Published:** 2011-10-26

**Authors:** Hooshang Hamidian, Samieh Fozooni, Asadollah Hassankhani, Sayed Zia Mohammadi

**Affiliations:** 1 Department of Chemistry, Payame Noor University (PNU), P.O. Box 19395-369776175-559, TehranKerman, Iran; Email: szmohammadi@yahoo.com (S.Z.M.); 2 Mining and Engineering Department of Zarand, Shahid Bahonar University, Kerman, 76169-14111, Iran; Email: s_fozooni@yahoo.com (S.F.); 3 Department Institute of Materials Environmental Science, International Center for Science and High Technology and Environmental Science, P.O. Box 76315-117, Kerman, Iran; Email: ahassankhani@gmail.com (A.H.)

**Keywords:** multicomponent reaction, nanosilica sulfuric acid, microwave irradiation, rice husk

## Abstract

A novel synthesis of triazolo[1,2-a]indazole-1,3,8-trione derivatives by reaction of urazole, dimedone and aromatic aldehydes under conventional heating and microwave irradiation and solvent-free conditions using silica nanoparticles prepared from rice husk ash as catalyst is described. The new method features high yields, multicomponent reactions and environmental friendliness.

## 1. Introduction

Rice husk is an abundantly available material rich in silica. It is a large-volume waste product of the rice milling industry in rice producing countries. New studies have produced silica nanoparticles from rice husk [1,2,3]. In recent years, silica nanoparticles have gained importance in scientific research due to their easy preparation and wide applicability as fillers, pharmaceuticals and also in the field of catalysis [4,5,6,7,8]. The high surface area of the nanoparticles is responsible for their catalytic activity.

Multicomponent reactions (MCRs) enable three or more reactive partners to be combined, either sequentially or simultaneously, in one pot, to give a target library that incorporates diversity simply by varying the constitution of the starting subsets. MCRs are economically and environmentally very advantageous because multi-step syntheses produce considerable amounts of waste, mainly due to complex isolation procedures after each step, often involving expensive, toxic and hazardous solvents [7,8]. Heterocyclic compounds occur widely in Nature and many are essential to life. Nitrogen-containing heterocyclic molecules constitute the largest portion of chemical entities which are part of many natural products, fine chemicals and biologically active pharmaceuticals vital for enhancing the quality of life [9]. Among a large variety of nitrogen-containing heterocyclic compounds, heterocycles containing a urazole (1,2,4-triazolidine-3,5-dione) moiety are of interest because they constitute an important class of natural and non-natural products, many of which exhibit useful biological activities and clinical applications [10,11]. Novel methods for preparing heterocycles containing a urazole moiety have attracted much interest in recent years [12,13,14,15,16,17]. Despite the available synthetic methods, there still exists a need for developing more efficient procedures, which would allow the ready synthesis of polycyclic urazole systems. We report herein a new method for the preparation of triazolo[1,2-a]indazole-trione derivatives using nanosilica sulfuric acid under thermal and microwave irradiation and solvent-free conditions. The experimental procedure for the reactions is remarkably simple and does not require the use of toxic or expensive organic solvents.

## 2. Results and Discussion

**Scheme 1:**
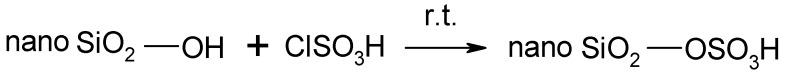
Conversion of nano-SiO_2_ into nanosilica sulfuric acid.

Rice husk samples used in this study were obtained from a rice mill. The samples were washed with distilled water to remove adhering soil and dust. Nanosilica particles have been synthesized by refluxing rice husk ash with 1 M NaOH and subsequently adjusting the pH using 1 M H_2_SO_4_ [3]. Nanosilica particles react with chlorosulfonic acid to give nanosilica sulfuric acid (Scheme 1). It is interesting to note that the reaction is easy and clean without any work-up procedure because HCl gas is immediately evolved from the reaction vessel [18].

**Scheme 2:**
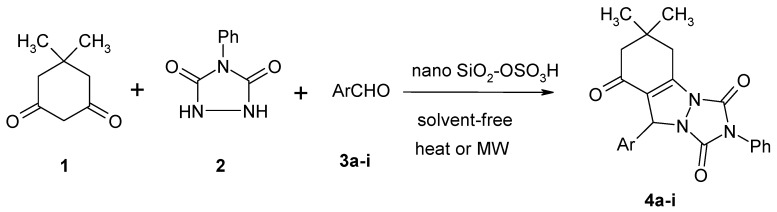
Synthesis of triazolo[1,2-a]indazole-triones catalyzed by nanosilica sulfuric acid under heating and microwave irradiation.

A mixture of dimedone (**1)**, urazole (**2)** and aromatic aldehydes **3** afforded 6,7-dihydro-6,6-dimethyl-2-phenyl-9-aryl-[1,2,4]-triazolo[1,2-a]indazole-1,3,8 (2*H*,5*H*,9*H*)-trione derivatives **4a–i** in good yields under thermal and microwave conditions in the presence of a catalytic amount of nanosilica sulfuric acid (Scheme 2).

**Scheme 3:**
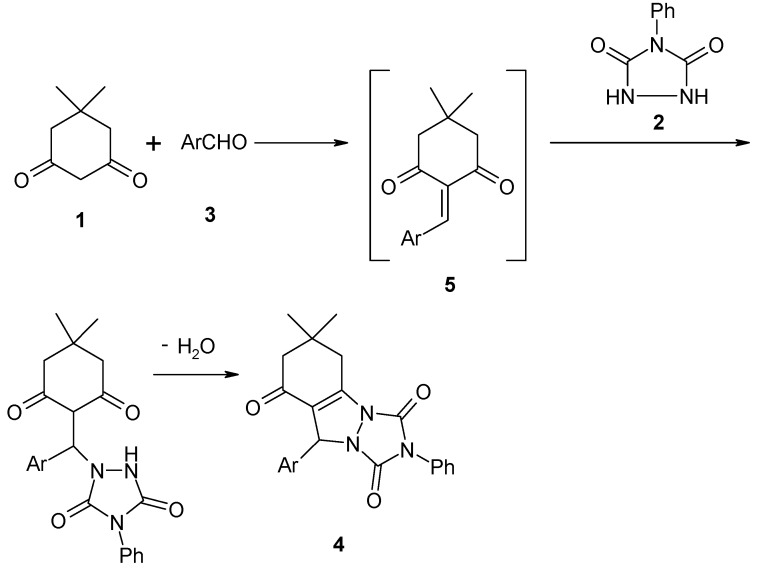
Michael-type addition of urazole to heterodiene and cyclization.

The formation of products **4a–i** can be rationalized by initial formation of heterodiene **5** by standard Knoevenagel condensation of dimedone (**1)** and aldehyde **3**. Subsequent Michael-type addition of urazole (**2**) to heterodiene **5** followed by cyclization afforded the corresponding products **4a–i** and water (Scheme 3).

We optimized the variables (MW power, amount of catalyst, time and temperature) in this reaction and the best results were obtained using 125 mg of nanosilica sulfuric acid and 400 W. The reaction of benzaldehyde was chosen as a model system under thermal conditions (Table 1) and MW irradiation (Table 2).

The results obtained by the two methods conventional heating (method A) and MW irradiation (method B) with the method of reference [19] were compared (Table 3). Compounds **4a–i** are stable solids whose structures were established by IR, ^1^H- and ^13^C-NMR spectroscopy, mass spectrometry and elemental analysis. The mass spectra of products **4a–i** displayed molecular ion peaks at appropriate values, which were consistent with the proposed 1:1:1 adduct of dimedone (**1**), urazole (**2**) and aldehyde **3**.

**Table 1:** Optimization of reactions under thermal conditions.

Entry	Catalyst (mg)	Time (min)	Temperature (°C)	Yield (%)
1	Silica nanoparticles (100)	30	80	-
2	Nanosilicasulfuric acid (80)	30	80	56
3	Nanosilicasulfuric acid (100)	30	80	67
4	Nanosilicasulfuric acid (125)	30	80	80
5	Nanosilicasulfuric acid (150)	30	80	80
6	Nanosilicasulfuric acid (125)	30	70	61
7	Nanosilicasulfuric acid (125)	30	90	80
8	Nanosilicasulfuric acid (125)	30	100	80
9	Nanosilicasulfuric acid (125)	25	80	64
10	Nanosilicasulfuric acid (125)	40	80	80

^a^ Isolated yield.

**Table 2:** Optimization of reactions under microwave irradiation (400 W).

Entry	Catalyst (mg)	Time (min)	Yield ^a^ (%)
1	Silica nanoparticles (125)	5	-
2	Nanosilicasulfuric acid (125)	3	52
3	Nanosilicasulfuric acid (125)	4	74
4	Nanosilicasulfuric acid (125)	5	92
5	Nanosilicasulfuric acid (125)	6	81

^a^ Isolated yield.

**Table 3:** Synthesis of triazolo[1,2-a]indazole-1,3,8-trione derivatives using nanosilica sulfuric acid obtained from rice husk.

Entry	Aldehyde	Product	Method A		Method B		M.P.(°C) [19]
			Time (min)	Yield ^a^ (%)	Time (min)	Yield ^a^ (%) ^b^	
1	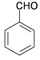	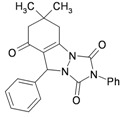	30	80	10	92 [78] *	189–190 [188–190] *
2	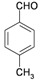	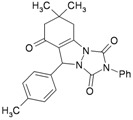	45	75	10	91 [79] *	161–163 [160–162] *
3	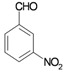	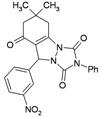	25	87	10	94 [83] *	125–126 [126–128] *
4	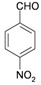	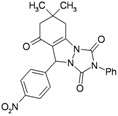	20	90	10	94 [81] *	173–175 [175–177] *
5	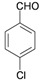	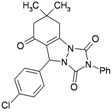	25	86	10	94 [88] *	169–171 [166–168] *
6	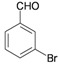	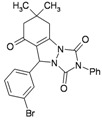	35	80	10	93 [81] *	175–177 [174–176] *
7	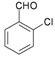	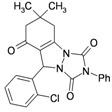	25	84	10	92 [79] *	171–172 [173–175] *
8	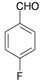	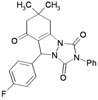	25	90	10	96 [90] *	105–106 [102–104] *
9	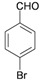	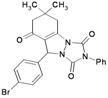	35	86	10	95 [80] *	185–186 [184–186] *

^a^ Yields refer to isolated and characterized pure products; ^b^ Yields obtained by method of reference [19]; * Compare with reference [19].

Nanosilica sulfuric acid obtained from rice husk is a good proton source in terms of convenience, cheapness, and easy production. The cheapness and availability of the reagents, easy procedure and facile work-up make this method attractive for the large- scale operations. Nanosilica sulfuric acid obtained from rice husk not only exhibits excellent activity in this one-pot reaction, but also simplifies recycling and reuse of the catalyst. The catalyst was separated by filtration and washed with ethanol, then it was activated at 80 °C under reduced pressure. This catalytic system retained its activity over six consecutive runs (Table 4). The catalytic activity of silica nanoparticles prepared from rice husk was compared with commercial silica nanoparticles, with both showing similar activity.

**Table 4:** Recyclability of nanosilica sulfuric acid prepared of rice husk.

Run	Yield ^a^ (%)	
	Method A	Method B
1	96	98
2	94	96
3	92	95
4	90	93
5	89	91
6	86	90

^a^ Isolated yield.

## 3. Experimental Section

### 3.1. General

Chemicals were purchased from the Merck company. Melting points were determined on a Thermo Fisher Scientific melting point apparatus and are uncorrected. Microwave reactions were performed with a Micro-SYNTH lab station reactor. All reactions were monitored by thin-layer chromatography (TLC) using Merck 60 silica gel F_254_ precoated glass-backed sheets. Silica nanoparticles were prepared by refluxing rice husk ash [3]. IR spectra were recorded with the MATTSON 1000 FT-IR Spectrophotometer. Nuclear magnetic resonance spectra were recorded on the BRUKER DRX- 500 AVANCE spectrometer using tetramethylsilane (TMS) as an internal standard. Mass spectra were obtained by SHIMADZU QP 5050 EX. Elemental analyses were performed by the Iranian Oil Company using a Heracus CHN-O-Rapid analyzer.The products were characterized by comparision of their spectral and melting point data with reference [19].

### 3.2. General Procedure (Method A)

A mixture of dimedone (**1**, 5 mmol), urazole (**2**, 5 mmol), aldehyde **3a–i** (6 mmol) and nanosilica sulfuric acid (125 mg) [18] was heated at 80 °C in a round-bottom flask for the appropriate time. After completion of reaction (monitored by TLC) the mixture was cooled to room temperature, then EtOAc (10 mL) was added to the mixture, which was filtered to remove the catalyst. The washing step was repeated twice. After evaporation of the solvent, the residue recrystallized from ethyl acetate/hexane (1:3) to afford pure product **4a–I** [19].

### 3.3. General Procedure (Method B)

In a high pressure Teflon reactor equipped with a magnetic stir bar and an optical fiber (for controlling the reaction temperature), a mixture of dimedone (**1**, 5 mmol), urazole (**2**, 5 mmol), aldehye **3a–i** (6 mmol) and nanosilica sulfuric acid (125 mg) was subjected to microwave irradiation at 80 °C (400 W) for the appropriate time (see Table 3) using a Micro-SYNTH lab station reactor. The mixture was cooled to room temperature, then EtOAc (10 mL) was added to the mixture which was filtered to remove the catalyst. The washing step was repeated twice. After evaporation of the solvent, the residue was recrystallized from ethyl acetate/ hexane (1:3) to afford pure product **4a–i**.

## 4. Conclusions

This method represents the first application of nanosilica particales prepared from rice husk as a powerful heterogeneous catalyst in organic synthesis and we have described an efficient, one-pot and simple method for the synthesis of triazolo[1,2-a]indazole-1,3,8-triones under solvent-free conditions using conventional heating and microwave irradiation.
